# Behavioral Alterations in Response to Fear-Provoking Stimuli and Tranylcypromine Induced by Perinatal Exposure to Bisphenol A and Nonylphenol in Male Rats

**DOI:** 10.1289/ehp.6961

**Published:** 2004-05-26

**Authors:** Takayuki Negishi, Katsuyoshi Kawasaki, Shingo Suzaki, Haruna Maeda, Yoshiyuki Ishii, Shigeru Kyuwa, Yoichiro Kuroda, Yasuhiro Yoshikawa

**Affiliations:** ^1^Department of Biomedical Science, Graduate School of Agricultural and Life Sciences, University of Tokyo, Tokyo, Japan; ^2^Core Research for Evolutional Science and Technology, Japan Science and Technology Agency, Saitama, Japan; ^3^Department of Psychology, Hoshi University, Tokyo, Japan; ^4^Department of Molecular and Cellular Neurobiology, Tokyo Metropolitan Institute for Neuroscience, Tokyo, Japan

**Keywords:** behavior, bisphenol A, fear, learning, monoamine, nonylphenol

## Abstract

The purpose of this study was to examine whether perinatal exposure to two major environmental endocrine-disrupting chemicals, bisphenol A (BPA; 0.1 mg/kg/day orally) and nonylphenol [NP; 0.1 mg/kg/day (low dose) and 10 mg/kg/day (high dose) orally] daily from gestational day 3 to postnatal day 20 (transplacental and lactational exposures) would lead to behavioral alterations in the male offspring of F344 rats. Neither BPA nor NP exposure affected behavioral characteristics in an open-field test (8 weeks of age), in a measurement of spontaneous motor activity (12 weeks of age), or in an elevated plus-maze test (14 weeks of age). A passive avoidance test (13 weeks of age) showed that both BPA- and NP-treated offspring tended to delay entry into a dark compartment. An active avoidance test at 15 weeks of age revealed that BPA-treated offspring showed significantly fewer avoidance responses and low-dose NP-treated offspring exhibited slightly fewer avoidance responses. Furthermore, BPA-treated offspring significantly increased the number of failures to avoid electrical unconditioned stimuli within 5-sec electrical shock presentation compared with the control offspring. In a monoamine-disruption test using 5 mg/kg (intraperitoneal) tranylcypromine (Tcy), a monoamine oxidase inhibitor, both BPA-treated and low-dose NP-treated offspring at 22–24 weeks of age failed to show a significant increment in locomotion in response to Tcy, whereas control and high-dose NP-treated offspring significantly increased locomotion behavior after Tcy injection. In addition, when only saline was injected during a monoamine-disruption test, low-dose NP-treated offspring showed frequent rearing compared with the control offspring. The present results indicate that perinatal low-dose BPA or NP exposure irreversibly influenced the reception of fear-provoking stimuli (e.g., electrical shock), as well as monoaminergic neural pathways.

Recently, there has been increasing concern about the exposure of the developing fetus to environmental endocrine-disrupting chemicals (EDCs). The disruption of cognitive function and various behavioral traits due to EDC exposure has been suspected (Schantz and Widholm 2001) because the development of the central nervous system (CNS) is highly regulated by endogenous hormones directly, including gonadal hormones, and by hormonally regulated events that occur early in development. The main purpose of this study was to examine whether perinatal exposure to two well-known environmental EDCs, bisphenol A (BPA) and nonylphenol (NP), can lead to behavioral alterations in the male offspring of F344 rats.

BPA (4,4′-isopropylidene-2-diphenol) is a high-production4volume chemical used in the manufacture of polycarbonate plastics, epoxy resins, and polyester resins. Worldwide production of BPA has increased and will most likely continue to increase in the future (Staples et al. 1998). Human exposure to BPA can occur via BPA-containing products included in certain baby bottles, food containers, resin-based food can linings, and dental sealants. Previous reports revealed that BPA had estrogenic (Gaido et al. 1997; Laws et al. 2000), and antiandrogenic (Sohoni and Sumpter 1998) activity in *in vitro* and *in vivo* assays. More recent studies have also identified BPA as an antiandrogen by a yeast two-hybrid system (Lee et al. 2003) and as an antagonist to thyroid hormone activity (Moriyama et al. 2002). These various activities of BPA might exert complicated adverse effects on CNS development because endogenous hormones at appropriate levels at certain limited developmental stages are essential for normal CNS development.

NP (4-nonylphenol) is another environmental EDC with week estrogenic activity (White et al. 1994). NP is used as an additive or surfactant in the manufacture of plastics, and it is a degradation product of nonylphenol polyethoxylates, which are widely used. NP has been shown to have equal or even more estrogenic activity than BPA in *in vitro* and *in vivo* assays (Laws et al. 2000). Although weak androgenic NP activity was identified by Sohoni and Sumpter (1998), a more recent study using a yeast two-hybrid system revealed the antiandrogenic effects of NP (Lee et al. 2003). NP may thus have different effects according to the experimental conditions of each assay system. It is therefore also possible that NP exerts a variety of activities under *in vivo* conditions.

There have been a number of reports suggesting the adverse effects of perinatal exposure to BPA on various behavioral traits in laboratory rodents. In mice, exposure to BPA during fetal development was shown to alter maternal behavior (Palanza et al. 2002) and enhance a methamphetamine-induced abuse state (Suzuki et al. 2003). In rats, alteration of sociosexual behavior (Farabollini et al. 2002), play behavior (Dessi-Fulgheri et al. 2002), and impulsive behavior (Adriani et al. 2003); reduced response to amphetamines (Adriani et al. 2003); and reduced behavioral sexual differentiation (Kubo et al. 2001, 2003) have been demonstrated after perinatal BPA exposure. In our previous report using F344 rats (Negishi et al. 2003b), perinatal exposure to 4 mg/kg/day BPA significantly affected the appropriate avoidance responses of offspring at 8 weeks of age in a shuttle-box avoidance test, suggesting that some alteration took place in response to fear-provoking stimuli; these responses are furthermore known to be controlled by the monoaminergic system (Gingrich 2002; Giorgi et al. 2003; Inoue et al. 1994). When these results are taken together, it appears that perinatal exposure to BPA can interfere with the development of monoaminergic systems, which might in turn be responsible for subtle behavioral changes.

In contrast, only a few studies have reported the effects of perinatal NP exposure on the behavioral traits of the offspring of experimental animals. Ferguson et al. (2000) demonstrated toxicity of NP to mothers and offspring, but found no alterations in open-field activity and running wheel activity in offspring after perinatal NP exposure. Latendresse et al. (2001) reported that the intake of a sodium solution was increased in offspring perinatally treated with NP (2,000 ppm in diet; > 200 mg/kg/day), but instead of focusing on CNS alterations, the authors’ focus was the relationship between this increased intake and the renal toxicity of NP. However, the possibility remains that NP at a much lower (≤ 10 mg/kg/day) dose alters certain cognitive functions and/or fine behavioral characteristics, including the response to fear-provoking stimuli, but without being associated with general motor dysfunction.

In the present study, we examined the adverse effects of low-dose (0.1 mg/kg/day) perinatal BPA or NP exposure on behavioral characteristics. To this end, we performed a series of behavioral tests: an open-field test, a measurement of spontaneous activity during a dark phase, a step-through passive avoidance test, an elevated plus-maze test, and a two-way shuttle-box avoidance test. In addition, in order to evaluate suspected alterations in the monoaminergic system, we investigated behavioral responses to tranylcypromine (Tcy), a monoamine oxidase inhibitor.

## Materials and Methods

### Animals and treatments.

Male and female F344/N rats were purchased from SLC (Sizuoka, Japan). The animals were maintained under controlled temperature (24 ± 1°C) and humidity (55 ± 5%), on a 12-hr light (09:00–21:00 hr):12-hr dark (21:00–09:00 hr) cycle. Food and water were freely available. After acclimatization for 1 week, female rats were placed with males. Vaginal smears were examined daily: a sperm-positive smear determined gestational day (GD)0. After detection, the pregnant dams were housed individually and were randomly assigned to an exposure condition (*n* = 10–11/condition). The dams were orally exposed to BPA (0.1 mg/kg/day; Tokyo Kasei Kogyo, Tokyo, Japan) or NP (0.1 or 10 mg/kg/day; Tokyo Kasei Kogyo) dissolved in corn oil, or to corn oil alone (vehicle control; 2 mL/kg/day) from GD3 until postnatal day (PND)20. Oral administrations of BPA and NP were performed by gavage. Because animals were trained to receive the feeding needle before mating, this procedure was not stressful. The dams were examined for clinical signs of toxicity and were weighed daily before dosing. After parturition (PND0), the pups were counted, weighed, and assigned to groups of six pups per litter, maintaining equivalent sex distributions when possible. Pups remained with their biological mother. Offspring were weighed and the body weights recorded on PND0, 3, 7, 10, 14, and 21 and again at 8 and 13 weeks of age. We included the mean weight of each littermate in the statistical analysis. Male pups were marked with ink for identification; On PND21, the marked male pups were gathered from different litters and housed together according to treatment group (7–8/cage). The dams were anesthetized with diethyl ether and then sacrificed by exsanguination; the body weights and organ weights (liver, kidney, spleen, and thymus) were then recorded.

We randomly selected one male pup per litter to undergo a series of behavioral tests (*n* = 9–10/group). The remaining male pups in the litter were subjected to the measurement of organ weights (liver, kidney, spleen, thymus, brain, and testis) at weaning (PND21) or at 8 weeks of age. Although male rats were usually housed in groups according to experimental treatment, rats were housed individually for some behavioral tests. At the end of each behavioral test, rats were again housed in a group according to treatment. In this study, we excluded female pups from behavioral tests because the estrous cycle in mature females affects various behavioral characteristics. When using female animals, it is important to consider the estrous cycle in evaluating the results of behavioral tests that require several consecutive days. This study was approved by the Animal Care and Use Committee of the Graduate School of Agricultural and Life Sciences, University of Tokyo.

### Open-field behavior test.

At 8 weeks of age, animals were subjected to an open-field test. Each subject was housed individually for 24 hr before the test. The open-field apparatus was a rectangular field (56 × 39 cm); none of the animals was familiar with this apparatus. Open-field behavior was recorded for 5 min by a video camera positioned above the apparatus during the dark phase (21:30–23:00 hr) under a low white light; responses were automatically analyzed by a computer-assisted system, which classified observed behavior as locomotion, rearing, or “other” behaviors.

### Spontaneous motor activity.

We measured spontaneous motor activity in 12-week-old male offspring using a Supermex (Muromachi Kikai, Tokyo, Japan) (Masuo et al. 1997). The Supermex consisted of a sensor monitor, which was mounted above the cage to detect changes in heat across multiple zones of the cage through an array of Fresnel lenses. The body heat radiated by the animal was detected with the sensor head of the monitor, which contained paired infrared pyroelectric detectors. In this manner, the system allowed the monitoring and counting of all spontaneous movements. Each animal was housed individually in the experimental cage—a differently arranged housing cage with food and water freely available—for 24 hr before the measurement to become accustomed to this experimental condition; spontaneous activity was then measured for about 12 hr (starting at 21:00 hr). All counts were automatically totaled and recorded in 2-min intervals. We defined “immobile time” as 2 min with no signal (count = 0).

### Passive avoidance test.

We conducted the step-through passive avoidance test when the animals were 13 weeks of age. The test was carried out during the light phase (13:00–17:00 hr), and each animal was housed individually during the test. The passive avoidance apparatus consisted of light and dark compartments. The first time each animal was placed in this apparatus, an electric foot shock (0.25 mA, 3 sec) was delivered to the animal through the grid floor just after the animal had completely left the light compartment for the dark compartment. We recorded the latency period required before each animal entered the dark compartment after having been placed in the light compartment. Twenty-four hours later, a retention trial (with no shock) was performed, and the latency period before entering the dark compartment was recorded. In addition to the traditional measure, we recorded the frequency and percentage of duration of poking into the dark compartment until the animal completely entered the dark compartment in the retention trial. If an animal failed to enter the dark compartment within 20 min, the test was terminated.

### Elevated plus-maze test.

The elevated plus-maze apparatus consisted of two open arms (50 × 10 cm) and two closed arms (50 × 10 cm, with 50-cm high walls) extending from a central square platform (10 × 10 cm); arms were arranged so that those of the same type were opposite each other. The apparatus was elevated 60 cm above the floor. At 14 weeks of age, each animal was placed in the central square facing an open arm during the dark phase (21:30–23:00 hr). We then recorded standard spatiotemporal factors for 5 min (i.e., the frequency of entries into the open arms and the closed arms was recorded, whereby “arm entry” was defined as moving the head into an open arm).

### Active avoidance test.

At 15 weeks of age, animals were subjected to an active avoidance test in a two-way shuttle-box (Muromachi Kikai, Tokyo, Japan) consisting of two compartments connected to each other by a hole in the wall; this test was carried out during the light phase (13:00–17:00 hr). Each animal was housed individually through the active avoidance test. Each animal was allowed to become accustomed to the shuttle-box apparatus for 5 min before every session; the animal was then subjected to 25 daily trials/session of avoidance conditioning in four consecutive sessions (acquisition test). For each trial, a 5-sec conditioned stimulus (CS), consisting of a buzzer and light, was followed by a 5-sec unconditioned stimulus (UCS), which included a scrambled shock of 0.2 mA delivered through the floor grid. In addition, on the day after the fourth session, each rat performed the extinction test, which is basically the avoidance text without the UCS. Each trial was separated by variable intertrial intervals (10–90 sec between trials; total of 1,250 sec/session). During the acquisition tests in sessions 1–4 and the extinction test, we recorded the percentage of correct avoidance responses, in which the animals moved to the other compartment of the shuttle box within a 5-sec CS in each block of 25 trials. To evaluate further behavioral characteristics in this procedure, we recorded the percentage of failures to avoid the stimulus within 5-sec UCS and the latency periods associated with both the CS and UCS throughout the four acquisition sessions.

### Monoamine-disruption test.

Disturbances of the monoaminergic system in the CNS were induced by a single intraperitoneal (i.p.) injection of Tcy (*trans*-2-phenylcyclopropyl-amine hydrochloride; Sigma-Aldrich, St. Louis, MO, USA). At 22–24 weeks of age, BPA- or NP-treated male offspring were subjected to the monoamine-disruption test. Before the monoamine-disruption tests, we determined the optimal dose of Tcy for the monoamine-disruption test in a different set of male F344 rats (*n* = 15) at 9 weeks of age. We injected (i.p.) Tcy solution in 0.9% saline at 0, 2, 5, and 10 mg/mL (1 mL/kg) at 16:00 hr (*n* = 4, 4, 4, and 3, respectively) and then measured spontaneous motor activity as described above. We confirmed that animals treated with 5 mg/kg Tcy showed a high increment of activity at 21:30 hr, when open-field behavior was recorded.

#### Saline challenge.

On the first day of the monoamine-disruption test, we injected 0.9% saline (1 mL/kg; i.p.) as a vehicle into each rat; 5.5 hr after the injection, we observed and recorded the behavior of the animals in the open-field apparatus for 4 min.

#### Tcy challenge.

On the day after the saline challenge, we injected 5 mg/kg Tcy (i.p.) into the same animal and recorded open-field behavior for 4 min, as described for the saline challenge. Behavioral analyses in the monoamine-disruption test were performed as described for the open-field test.

### Statistical analyses.

We conducted statistical analyses using StatView, Version 5.0 (SAS Institute, Cary, NC, USA). We analyzed the effects of perinatal BPA or NP exposure on maternal body weight increase and the body weight of male offspring by analysis of variance (ANOVA) with one between-subject factor (treatment) and one repeated-measures factor (days). The number of total, male, and female pups;, the organ weights of dams at weaning; and the organ weights of male offspring at PND21 and at 8 weeks of age were analyzed by one-way ANOVA. Behavioral measurements were analyzed by one-way ANOVA, except for the percentages of correct avoidance in the shuttle-box avoidance test, which were assessed by repeated measures of ANOVA over days (sessions). In the analysis of latency in the passive avoidance test, data processed through logarithmic transformations were used for the ANOVA because of their significantly inappropriate distributions with respect to the normal distributions. In each statistical analysis, the effects of BPA and NP exposure were analyzed with respect to the control in the same ANOVA. When the ANOVA produced significant results, we then performed the post hoc Fisher’s protected least-significant difference test for comparisons between groups. The significance level for all tests was set at *p* < 0.05.

## Results

### Maternal toxicity and reproductive results.

Oral exposure to BPA or NP showed no statistically significant effect on maternal body weight increase during pregnancy and lactation or on the number of total, male, and female pups (data not shown). All dams in this study delivered their offspring on GD22. There was no significant effect of 40-day exposure to BPA or NP on either body weight or organ weights (data not shown).

### Development of male offspring.

Perinatal exposure to BPA or NP had no significant effect on either body weight gain or organ weights of male offspring on PND21 or at 8 weeks of age (data not shown). No male offspring died during the course of the study (> 25 weeks of age).

### Open-field test.

In the open-field test, neither BPA nor NP exposure significantly affected the percentage of locomotion [*F*_(3,32)_ = 0.271, *p* > 0.5] or the number of rearings [*F*_(3,32)_ = 0.189, *p* > 0.5; data not shown].

### Spontaneous motor activity.

To assess general motor activity under nonstress conditions, we recorded spontaneous motor activity of male offspring. Neither BPA nor NP exposure had any effect on the rhythm of activity, the total counts of activity [*F*_(3,32)_ = 0.554, *p* > 0.5], or the immobile time [*F*_(3,35)_ = 0.078, *p* > 0.5] during the 12-hr dark phase (data not shown).

### Passive avoidance test.

**Figure 1:**
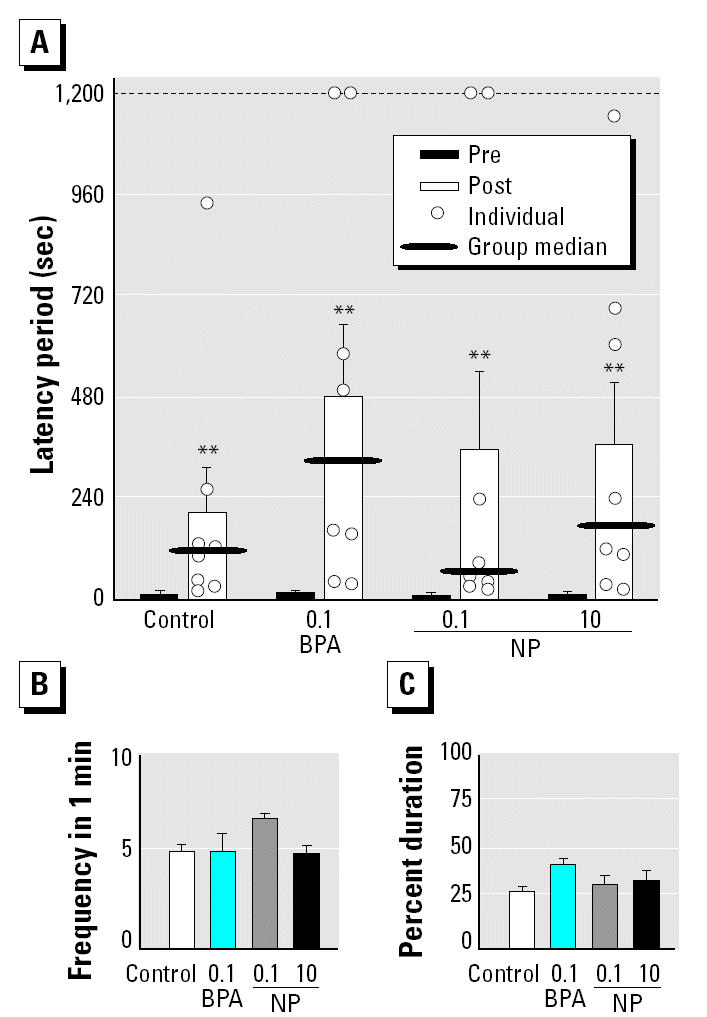
Effect of perinatal exposure (mean ± SE) to BPA or NP (mg/kg/day) on the behavioral characteristics in a passive avoidance test (*n* = 8/group). Abbreviations: Post, latency during the retention trial; Pre, latency on shock-presenting day. (*A*) The latency period until the animals completely entered the dark compartment. (*B*) The frequency of poking into a dark box until complete entrance. (*C*) The percent duration of poking into a dark box until complete entrance.
**p < 0.01 compared with Pre for same treatment.

On shock-presenting day of the passive avoidance test, the subjects readily entered the dark compartment (< 30 sec). During the retention trial 24 hr after shock presentation, ANOVA [*F*_(7,63)_ = 12.174, *p* < 0.001] and multiple comparisons revealed that the subjects showed significant hesitation (*p* < 0.01) to enter the dark compartment compared with the short latency during shock presentation in all of the experimental groups (Figure 1A). Although both BPA- and NP-treated groups tended to remain in the light compartment longer than the control offspring, there was no significant difference in latency periods during the retention trial among the experimental groups. Neither the frequency of poking into the dark [*F*_(3,28)_ = 1.166, *p* > 0.1; Figure 1B] nor the percentage of duration of poking into the dark [*F*_(3,28)_ = 1.919, *p* > 0.1; Figure 1C] during the retention trial was affected by chemical exposure.

### Elevated plus-maze test.

Neither BPA nor NP exposure significantly altered the frequency of entering the open arms [*F*_(3,27)_ = 0.571, *p* > 0.5] or the closed arms [*F*_(3,27)_ = 0.139, *p* > 0.5] of the elevated plus-maze test, although the frequency of entering the open arms was slightly higher in the BPA-treated group than in the controls (data not shown).

### Active avoidance test.

**Figure 2:**
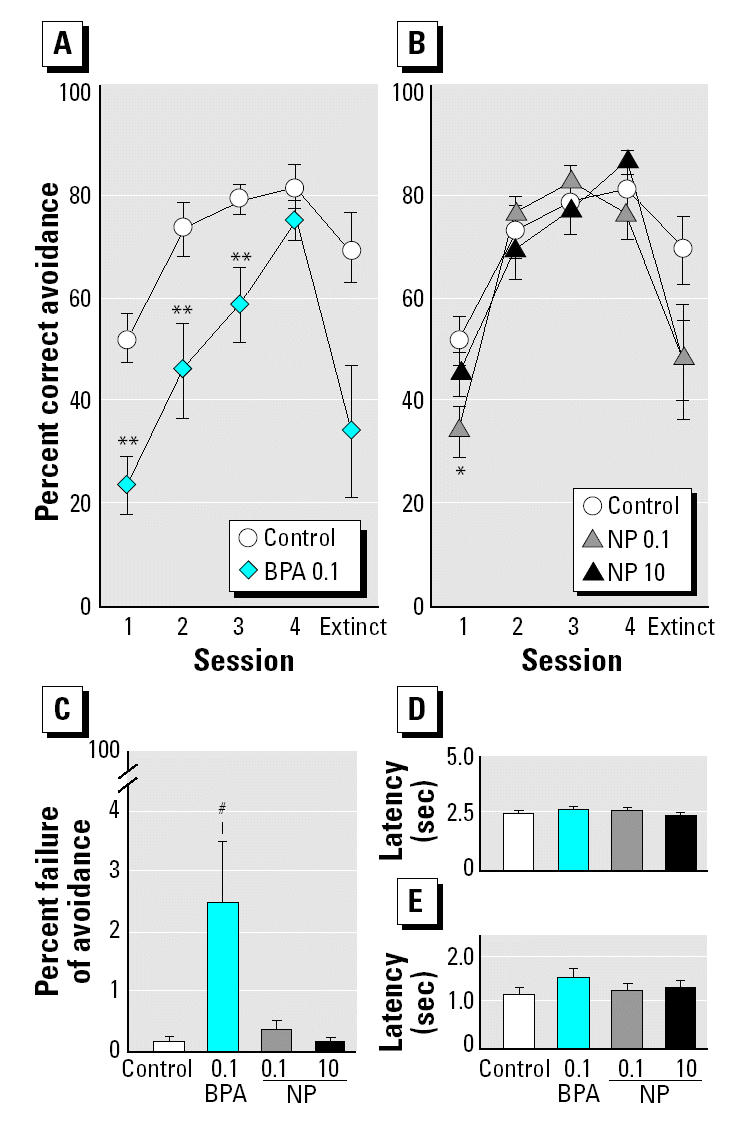
Effects of perinatal exposure to BPA or NP (mg/kg/day) on behavioral characteristics in a shuttle-box avoidance test (mean ± SE; *n* = 9–10/group). Avoidance learning curves of male offspring perinatally exposed to BPA (*A*) or NP (*B*). (*C*) Percentage of failure of avoidance when an electrical shock was presented for 5 sec among 100 trials of four sessions. Length of latency period associated with a CS (*D*) and a UCS (*E*) in four sessions.
*p < 0.05;
**p < 0.01; and ^#^*p* < 0.001 compared with control.

BPA and low-dose NP exposure significantly affected the avoidance responses of the male offspring in the active avoidance test. Repeated-measures one-way ANOVA showed a significant effect of chemical exposure [*F*_(3,35)_ = 5.724, *p* < 0.01] and number of sessions [*F*_(3,105)_ = 107.322, *p* < 0.0001], as well as a significant interaction between chemical exposure and the number of sessions [*F*_(9,105)_ = 3.536, *p* < 0.001]. One-way ANOVAs and post hoc multiple comparisons for each session indicated significantly fewer avoidance responses in BPA-treated offspring at the first, second, and third sessions than in the control offspring (Figure 2A). Low-dose NP-treated offspring showed a lower avoidance rate than the control offspring, but only in the first session (Figure 2B). In the fifth extinction session (i.e., without electrical shocks as the UCS), the BPA- and NP-treated offspring showed slightly less correct avoidance behavior than the control offspring, although the effect of chemical exposure, as determined by one-way ANOVA, was not statistically significant [*F*_(3,35)_ = 0.571, *p* = 0.104]. One-way ANOVA indicated a significant effect of chemical exposure and that the frequency of failure of avoidance within 5 sec of shock presentation—in which one-way ANOVA indicated a significant effect of chemical exposure [*F*_(3,35)_ = 3.700, *p* < 0.05]—in BPA-treated offspring was significantly higher (*p* < 0.001) than that in the control offspring; low-dose NP-treated offspring showed a similar tendency (Figure 2C). We found no significant effect of chemical exposure on the mean of the latency periods associated with CS [*F*_(3,35)_ = 0.722, *p* > 0.5; Figure 2D] and UCS [*F*_(3,35)_ = 1.186, *p* > 0.1; Figure 2E] in 100 trials of four sessions.

### Monoamine-disruption test.

**Figure 3:**
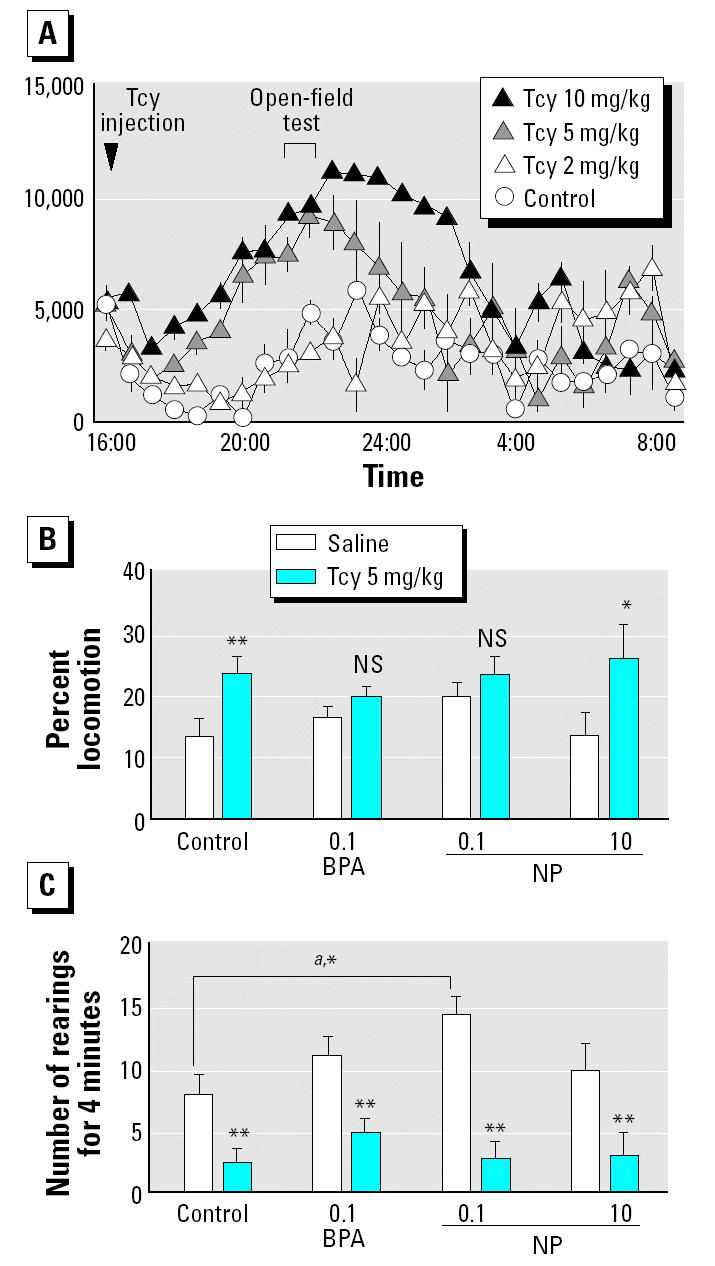
Effects of perinatal exposure to BPA or NP (mg/kg/day) on behavior in the monoamine-disruption test 5.5 hr after Tcy treatment. NS, not significant. (*A*) Locomotor activity (mean ± SE) after a single injection with 2, 5, or 10 mg/kg/day Tcy (*n* = 3–4/group). (*B*) Effect of perinatal BPA and NP on Tcy (5 mg/kg)-induced increases in locomotion behavior in an open-field apparatus (mean ± SE; *n* = 7–9/group). (*C*) Effects of perinatal BPA and NP on Tcy (5 mg/kg)-induced suppression of rearing in an open-field apparatus (mean ± SE; *n* = 7–9/group). ***^a^***The bracket indicates the comparison of offspring exposed to BPA and low-dose NP in the saline challenge.
*p < 0.05.
**p < 0.01 compared with saline control.

Tcy injection led to a large, slow increase in motor activity at 5 and 10 mg/kg compared with the saline control, although 2 mg/kg Tcy did not induce an increase (Figure 3A). We confirmed that the animals administered 5 mg/kg Tcy showed a high increase of activity 5.5 hr after injection, which corresponded to the schedule for the monoamine-disruption test. One-way ANOVA and post hoc tests about locomotion behavior [*F*_(7,58)_ = 2.498, *p* < 0.05] and the number of rearing behaviors [*F*_(7,58)_ = 9.629, *p* < 0.01] yielded the following results. In the monoamine-disruption test, control and high-dose NP-treated offspring showed a significant increase in locomotion behavior resulting from Tcy injection (*p* < 0.01; Figure 3B). However, BPA-treated or low-dose NP-treated offspring failed to show a clear increase in locomotion (*p* > 0.1). Tcy also caused significant decreases in the number of rearing behaviors in all experimental groups (Figure 3C) in the monoamine-disruption test. When only saline was administered, low-dose NP-treated offspring showed a significantly increased number of rearing behaviors, compared with those of the control offspring in the saline challenge; in addition, the BPA-treated offspring also appeared to have a similar tendency (*p* < 0.1), which was abolished by monoamine disruption by Tcy (Figure 3C).

## Discussion

In the present study, we carried out a series of behavioral tests and demonstrated the subtle and complex functional effects of perinatal exposure to BPA and NP on the behavior of male rat offspring. Behavioral alterations by perinatal exposure to BPA and NP were detected only in specific challenges involving fear-provoking stimuli and pharmacologic disruption of monoaminergic system, whereas spontaneous explorative behavior and responses to novelty were not affected by the exposure to these chemicals.

Evaluation of the toxicity of BPA as well as that of NP on maternal body weight, parturition, maternal organ weights at weaning (PND21), and general development (body weight and organ weights) of the offspring confirmed that there were no adverse effects of BPA at 0.1 mg/kg/day or of NP at 0.1 and 10 mg/kg/day, which was consistent with the findings of previous reports (Ferguson et al. 2000; Kwon et al. 2000).

In the present study, we detected no statistically significant alterations by BPA or NP in the spontaneous activity and behaviors of rats in the open-field test at 8 weeks of age and in the elevated plus-maze test at 14 weeks of age. This suggests that these chemical exposures induce no severe abnormalities in general behavior.

In the passive avoidance test, offspring perinatally exposed to BPA or NP seemed to be more sensitive to fear-inducing shock than were the control offspring, which might have led to the somewhat stronger retention in the chemical-treated groups; however, such changes were statistically ambiguous because of the large individual differences in the experimental conditions used in this study.

In the active avoidance test, BPA and low-dose NP showed clear or partial adverse effects on behavior, respectively. In particular, BPA-treated offspring may have been less able to learn than the control offspring in terms of causality. Low-dose NP-treated offspring were also affected to some extent. Although the possibility remains that BPA-treated offspring were more insensitive to electrical shock than were the control offspring, the slight elongation of the latency period in the passive avoidance test would have excluded this possibility. If BPA-treated offspring had found the electrical stimuli less fear-provoking and/or painful, they would have entered the dark compartment more quickly than the control offspring in the passive avoidance test. It would also be unlikely that less learning took place as a result of motor dysfunction or sensory abnormality because there was no alteration in the spontaneous activity during the dark phase and in the duration of locomotion in the open-field test, as well as in the latency periods associated with CS and UCS in the active avoidance test. When electrical shock was presented as a UCS, BPA-treated offspring failed to enter the opposite compartment within 5 sec more frequently than the control offspring, and NP-treated offspring showed a similar tendency. BPA-treated offspring tended to stiffen in the corner of the box during the UCS, and these animals appeared to stop avoiding the UCS more often than did the control offspring, as determined by direct observation (data not shown). It is possible that excessive fear of the UCS would interfere with the smooth progression of avoidance learning. Perinatal BPA exposure may render male offspring exceedingly vulnerable to intolerable levels of fear. Interestingly, this hypothesis may be supported by a previous study (Aloisi et al. 2002), which indicated that perinatal BPA exposure increased the sensitivity of the central neural pathways for nociception in male offspring. Farabollini and colleagues reported the details of various behavioral changes observed due to perinatal exposure to BPA at 0.04 or 0.4 mg/kg/day in rats (Adriani et al. 2003; Aloisi et al. 2002; Dessi-Fulgheri et al. 2002; Farabollini et al. 1999, 2002). In the present study we also provided new evidence of the behavioral adverse effects of perinatal exposure to BPA at a low dose of 0.1 mg/kg/day on electrical UCS-related responses in an active avoidance test.

In the monoamine-disruption test, Tcy-induced increases in locomotion were significantly less marked in BPA-treated and low-dose NP-treated offspring, but not in the high-dose NP-treated offspring, compared with the control offspring. This is the first study reporting behavioral alterations due to perinatal BPA and NP exposure shown by responses to the disruption of monoaminergic systems, with Tcy having clear and straightforward pharmacologic effects as a monoamine oxidase inhibitor. Previous studies have reported changes after BPA exposure in a mouse model of psycho-stimulant abuse (methamphetamine) (Suzuki et al. 2003) and in a rat model of an increment in activity by amphetamine (Adriani et al. 2003), which have complicated pharmacologic effects on CNS function. It is possible that BPA-treated and low-dose NP-treated offspring might be insensitive to monoamines overflowing in excess into the extrasynaptic space. Such animals might show abnormal expressions of each type of monoamine receptor (dopamine, serotonin, and noradrenaline receptors, including the subtypes of each receptor class) or monoamine oxidase in certain region(s) of the CNS. Further investigations considering each of the monoamergic systems (dopaminergic, serotoninergic, and noradrenalinergic) are likely to produce more insight into the mechanism of traces induced by perinatal BPA and NP exposure in the CNS. Although monoamine disruption by Tcy significantly reduced the number of rearing behaviors, the response to Tcy was not influenced by perinatal chemical exposure. There was a discrepancy between the results of behavior in the open-field apparatus at 8 weeks of age and > 22 weeks of age. When the animals were > 22 weeks of age, the observed significant increase in the number of rearing behaviors suggested that low-dose NP-treated offspring might have experienced less anxiety than the control offspring in the open-field apparatus. The results furthermore suggested that these behavioral alterations caused by perinatal low-dose NP exposure might appear only at a stage of advanced age, that is, at a time when rats are relatively slow in their movements and rarely show rearing compared with juveniles. Further investigations will be required in this regard. In any case, the effective dose of NP from a neurobehavioral standpoint is much lower than the dose associated with general physical toxicity, as observed in the case of BPA in our previous study of that substance (Negishi et al. 2003b).

We cannot address differences in sexes in the present study because we limited this behavioral study to the male offspring; our primary goal was to detect behavioral alteration by perinatal BPA exposure in male offspring. However, studies in rats by Kubo et al. (2001, 2003) have demonstrated that perinatal exposure to BPA removed the differences between the sexes in the volume of locus ceruleus and in behavioral characteristics. In addition, some studies have reported sex differences in the effects of BPA (Dessi-Fulgheri et al. 2002; Farabollini et al. 1999,  2002). Further experiments using the active avoidance test and the monoamine-disruption test on both male and female offspring would be informative.

In the present study, one animal per litter sequentially underwent all of the behavioral tests. It is possible that an experience in an earlier behavioral test influenced the results of the subsequent behavioral tests. For example, a painful experience immediately after exploratory behavior in the passive avoidance test might interfere with the behavioral propensity in the elevated plus-maze test. However, we believed that using sequential behavioral tests in the same animal would not obstruct the evaluation of effects of perinatal exposure to chemicals because all of the animals in the four treatment groups experienced the stimuli.

In summary, perinatal exposure to BPA and NP, both at 0.1 mg/kg/day, affected the extent of shock-related behavior and affected responses to the disruption of the mono-aminergic system, although the direct mechanisms of these alterations remain unclear at present. Moreover, the neurobehavioral toxicity of both BPA and NP may be out of proportion with the *in vitro* and *in vivo* estrogenic potency of these compounds determined by certain simple assay systems. It may be useful to consider other potencies and/or metabolites of BPA and NP (Moriyama et al. 2002; Yoshihara et al. 2001) in addition to their weak estrogenic activity. We suggest that there may be a causal relationship between behavioral alterations in response to fear-provoking stimuli and abnormality in the mono-aminergic system because both dopamine and serotonin play important roles in the processing of fear-provoking and/or stressful stimuli in the CNS (Gingrich 2002; Giorgi et al. 2003; Inoue et al. 1994). In our recent study using primary cultured neurons (Negishi et al. 2003a), BPA and NP inhibited staurosporine-induced neuronal cell death, interfering with caspase-3 activation. BPA and NP may, in this manner, disrupt programmed neuronal cell death during development, which would irreversibly lead to an abnormal neural network—including the monoaminergic system—and cause behavioral abnormalities in adulthood.

## Conclusion

We conclude that perinatal BPA and NP exposure, even at slightly higher doses than those associated with environmental exposure in humans, had adverse behavioral effects on rats, especially when the animals were forced to avoid fear-provoking stimuli such as electrical shocks. Perinatal exposure to BPA and NP disrupted the reception of intolerable stress, which may be due to the alterations in monoaminergic system.
